# Illness Perceptions and Medication Nonadherence to Immunosuppressants After Successful Kidney Transplantation: A Cross-Sectional Study

**DOI:** 10.3389/ti.2022.10073

**Published:** 2022-02-07

**Authors:** Yiman Wang, Denise M. J. Veltkamp, Paul J. M. van der Boog, Marc H. Hemmelder, Friedo W. Dekker, Aiko P. J. de Vries, Yvette Meuleman

**Affiliations:** ^1^ Department of Clinical Epidemiology, Leiden University Medical Center, Leiden, Netherlands; ^2^ Department of Internal Medicine, Division of Nephrology, Leiden University Medical Center, Leiden, Netherlands; ^3^ Transplant Center, Leiden University Medical Center, Leiden, Netherlands; ^4^ Department of Internal Medicine, Division of Nephrology, Maastricht University Medical Center, Maastricht, Netherlands; ^5^ CARIM School for Cardiovascular Research, University Maastricht, Maastricht, Netherlands

**Keywords:** kidney transplantation, adult, illness perceptions, immunosuppressants, medication nonadherence

## Abstract

**Background:** Medication nonadherence to immunosuppressants is a well-known risk factor for suboptimal health outcomes in kidney transplant recipients (KTRs). This study examined the relationship between illness perceptions and medication nonadherence in prevalent Dutch KTRs and whether this relationship depended on post-transplant time.

**Methods:** Eligible KTRs transplanted in Leiden University Medical Center were invited for this cross-sectional study. The illness perceptions and medication nonadherence were measured *via* validated questionnaires. Associations between illness perceptions and medication nonadherence were investigated using multivariable logistic regression models.

**Results:** For the study, 627 participating KTRs were analyzed. 203 (32.4%) KTRs were considered nonadherent to their immunosuppressants with “taking medication more than 2 h from the prescribed dosing time” as the most prevalent nonadherent behaviour (*n* = 171; 27.3%). Three illness perceptions were significantly associated with medication nonadherence: *illness identity* (adjusted odds ratio [OR_adj_] = 1.07; 95% confidence interval [CI], 1.00–1.14), *concern* (OR_adj_ = 1.07; 95%CI,1.00–1.14), and *illness coherence* (OR_adj_ = 1.11; 95%CI,1.01–1.22). The relationships between illness perceptions and medication nonadherence did not differ depending on post-transplant time (*p*-values ranged from 0.48 to 0.96).

**Conclusion:** Stronger negative illness perceptions are associated with medication nonadherence to immunosuppressants. Targeting negative illness perceptions by means of psychoeducational interventions could optimize medication adherence and consequently improve health outcomes in KTRs.

## Graphical Abstract

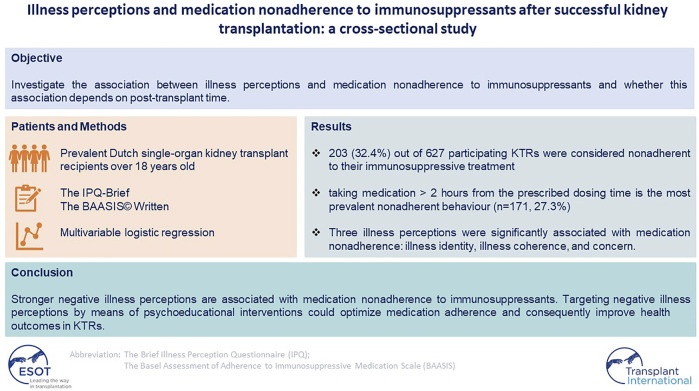

## Introduction

Successful kidney transplantation requires strict adherence to chronic immunosuppressive regimens (1). Failure to take immunosuppressants as prescribed has been identified as a risk factor for adverse clinical outcomes among kidney transplant recipients (KTRs), including graft loss and reduced patient survival (2, 3). Butler et al. reported a seven-fold higher odds of graft failure in nonadherent KTRs than in adherent KTRs (2). Furthermore, persistent medication nonadherence can lead to increased individual medical costs (4). Despite the obvious negative impact, medication nonadherence in KTRs remains substantial, with a broadly consistent prevalence of 20% or higher (1, 5).

Leventhal’s widely-used Common Sense Model (CSM) of Self-regulation provides us with explanations for patients’ behaviour when facing health threats and may aid our understanding of the behavioural mechanism explaining medication nonadherence (6). According to the CSM, patients’ illness perceptions directly influence their coping behaviour (e.g., medication adherence) with the medical condition; thereafter, they appraise the effect of such behavioural adaptions and the result of the appraisal therof can shape their illness perceptions (6). Consequently, illness perceptions—referring to patients’ appraisal and understanding of their medical condition—are considered a potential intervention target to improve coping behaviours and subsequent health outcomes.

Previous studies have shown that illness perceptions are associated with various outcomes in patients with chronic conditions, including chronic kidney disease (7–10). In non-KTRs (e.g., patients with hypertension), stronger positive illness perceptions have also been found associated with better medication adherence (11). However, very few studies have shed light on illness perceptions and their associations with medication nonadherence in patients after kidney transplantation, and the existing studies found inconsistent results: Cossart et al. (12) found stronger positive perceptions (i.e., illness coherence) in adherent KTRs, while Massey et al. (13) described a downward trend in medication adherence with improved illness perceptions over time. Therefore, further studies are necessary to understand the influence of illness perceptions on medication nonadherence and to develop effective patient-centered interventions to improve medication adherence in this KTR population.

Finally, the dynamic nature of the self-regulation process is an important feature of the CSM, which suggests that illness perceptions can change throughout the course of a disease (14, 15). A previous study has detected changes in certain illness perceptions in KTRs within 1.5 years after transplantation (13). It is reasonable to speculate that the relief after successful kidney transplantation may positively impact illness perceptions in the short term; however, in the long term, illness perceptions may change due to change in the experience of immunosuppressant-related side effects. Until now, little is known about whether such dynamic feature of KTRs’ illness perceptions also plays a role in medication adherence. Therefore, in this study, we will investigate the influence of illness perceptions on medication nonadherence to immunosuppressants among prevalent Dutch KTRs and explore whether such associations differ depending on the time since their kidney transplant.

## Patients and Methods

For the reporting of this study, we followed the STrengthening the Reporting of OBservational studies in Epidemiology (STROBE) guideline (16).

### Study Design and Study Population

This study was conducted in Leiden University Medical Center (LUMC) from 1 October 2020 to 30 October 2020. KTRs who met the following criteria were invited to participate in this study: 1) adult KTRs transplanted before 1 April 2019 in LUMC with a functioning graft; 2) the last visit in LUMC took place after 31 December 2010; and 3) patients with a sufficient understanding of the Dutch language. To avoid overburdening of patients, we did not invite patients transplanted after April 2019 as they were already involved in a longitudinal study to measure patient-reported outcomes after kidney transplantation routinely. We excluded patients whose last visit in LUMC was before 31 December 2010 to have more easily accessible administrative and clinical data.The questionnaires used in our study were sent to patients *via* postal service or email along with an informed consent form to use the collected data for research purposes. The questionnaires measured medication adherence and illness perceptions, and collected data about patients’ education level, marital status, and employment status at the time of the study. A reminder email was sent to patients with a known email address if they did not respond within 7 days after the first invitation. The institutional review board of LUMC for non-WMO research (i.e., research not subjected to the Medical Research Involving Human Subjects Act [WMO]) approved this study. The study was conducted following the national guidelines for medical scientific research (17).

### Medication Nonadherence

Self-reported medication adherence to immunosuppressants was measured using a commonly used and validated questionnaire, the Basel Assessment of Adherence to Immunosuppressive Medication Scale (BAASIS© Written) (18). The questionnaire contains four questions to measure medication adherence in the implementation phase (i.e., issue with taking, changed timing, drug holidays, and dose reduction). Each question asks the occurrence of the medication-taking behaviour (yes or no) and the frequency of corresponding nonadherent behaviour (i.e., once a month, once every 2 weeks, every week, more than once a week, and every day) in the past 4 weeks prior to the measurement. Regardless of the frequency, any “yes” to the above four questions implied medication nonadherence to immunosuppressants. The reporting of medication adherence followed the ESPACOMP Medication Adherence Reporting Guidelines (EMERGE) checklist (19).

### Illness Perceptions

The following eight illness perceptions were measured on a 0-to-10 response scale using the commonly used and validated questionnaire, the Brief Illness Perception Questionnaire (Brief-IPQ) (20): *consequences*, *timeline*, *personal control*, *treatment control*, *illness identity*, *concern*, *illness coherence,* and *emotional response*. In this study, we omitted illness perception domain *cause* from our analysis as the cause of kidney disease is very heterogeneous (7). To facilitate interpretation, we recoded the scores of three perceptions (i.e., *personal control*, *treatment control,* and *illness coherence*) in such a way that for all perceptions, a higher score indicated more negative illness perceptions (e.g., a higher score of treatment control now implies a lower belief of patients in that the treatment they receive can relieve or cure their illness).

### Sociodemographic and Clinical Characteristics

Data on sociodemographic and clinical characteristics were collected *via* questionnaires or from patients’ medical records, including age at transplantation, age at study participation, sex, socioeconomic status (SES), education level, marital status, number of transplantation, primary kidney disease, donor type (living donor and deceased donor), pre-emptive kidney transplantation, time since kidney transplantation (i.e., post-transplant time), body mass index (BMI), comorbidities, and type of immunosuppressants at study. The SES of study participants was obtained by linking the four digits of their postcode with the latest SES-score per postcode area reported by the Netherlands Institute for Social Research; the SES was divided into three groups: low, medium, and high (21). Primary kidney disease (PKD) was classified into eight categories: congenital and hereditary kidney disease, cystic kidney disease, diabetes mellitus, glomerulonephritis, renal vascular disease, interstitial nephritis/pyelonephritis, other diseases, and unknown ontology (22). Data about comorbidities at transplantation were collected. Comorbidities were indicated by a history of diabetes mellitus, cardiac event, vascular event, and cerebrovascular event before the study. Post-transplant time was ategorized into three groups: ≤5 years, 5–15 years, and >15 years. The most recent BMI was also collected, with the average time between BMI measurement and study participating being approximiatly 1 year (mean = 12.5 months; SD = 13.7 months).

### Statistical Analysis

Continuous variables were presented as mean with standard deviation (SD) if normally distributed and as median with interquartile range (IQR) if not normally distributed. Count (percentage) was used for categorical variables. Medication adherence and illness perceptions were described in the total study population and in subgroups stratified by post-transplant time. Multivariable logistic regression models were employed to analyse the impact of each separate illness perception on medication adherence while adjusting for potential confounders, including age at study participation, sex, SES, marital status, education level, employment status, donor type, number of transplantation, PKD, comorbidities, and post-transplant time. The interaction term “post-transplant time (categorical) * illness perception” was added to evaluate whether the influence of individual illness perception on medication nonadherence differed depending on post-transplant time. A variable “IPQ score/n” was used in the logistic regression models to assess the risk of medication nonadherence with n increments in IPQ-score (i.e., one or two increments on a 11-point scale).

Missing values were considered “missing at random” and were imputed with 10-folds multiple imputation (23). In addition to the variables with missing values (see Table 1), variables used for multiple imputation included illness perceptions, medication adherence, and other variables adjusted for in the logistical regression model. Abnormally distributed continuous variables were log-transformed for imputation. As sensitivity analyses, we repeated all analyses but now excluded comorbidities and BMI from the multivariable model due to a relatively high percentage of missing values. The patient characteristics of responders and nonresponders are presented in Supplementary Table S1. *P*-value < 0.05 was considered significant. We used SPSS software version 25.0. (IBM, Armonk, NY, United States) for all analyses.

**TABLE 1:** Patient characteristics of the total study population and stratified by categories of post-transplant time.

Characteristic	Total (*n* = 627)	Post-transplant time		
		<5 years (*n* = 158)	5–15 years (*n* = 312)	>15 years (*n* = 157)
Mean age (SD) at study, yr	61.4 (11.3)	58.0 (11.9)	61.8 (11.5)	63.9 (9.3)
Age structure at study, n (%)				
18∼39	31 (4.9)	14 (8.9)	15 (4.8)	2 (1.3)
40∼59	233 (37.2)	68 (43.0)	114 (36.5)	51 (32.5)
60∼79	350 (55.8)	76 (48.1)	176 (56.4)	98 (62.5)
80∼	13 (2.1)	0 (0)	7 (2.2)	6 (3.8)
Mean (SD) age at KT, yr	50.0 (13.1)	54.9 (11.8)	52.5 (11.8)	40.0 (11.5)
Median (IQR) time after KT, yr	9.0 (10.2)	3.1 (1.8)	9.0 (4.8)	20.7 (11.3)
Female, n(%)	233 (37.2)	53 (33.5)	124 (39.7)	56 (35.7)
SES, n(%)^a^				
Low	64 (10.2)	22 (13.9)	26 (8.3)	16 (10.2)
Middle	397 (63.3)	101 (63.9)	200 (64.1)	96 (61.1)
High	161 (25.7)	34 (21.5)	83 (26.6)	44 (28.0)
Marital status, n(%)				
Single/separated	160 (25.5)	53 (33.5)	71 (22.8)	36 (22.9)
Married/living together	467 (74.5)	105 (66.5)	241 (77.2)	121 (77.1)
Education				
Low	52 (8.3)	12 (7.6)	22 (7.1)	18 (11.5)
Middle	215 (34.3)	52 (32.9)	107 (34.3)	56 (35.6)
High	360 (57.4)	94 (59.5)	183 (58.7)	83 (52.9)
Employment, n(%)				
Employed	291 (46.4)	83 (52.5)	142 (45.5)	66 (42.0)
Unemployed	69 (11.0)	24 (15.2)	32 (10.3)	13 (8.3)
Retired/Student	267 (42.6)	51 (32.3)	138 (44.2)	78 (49.7)
Primary Kidney Disease, n(%)^a^				
Congenital/hereditary kidney disease	15 (2.4)	0 (0)	8 (2.6)	7 (4.5)
Cystic kidney disease	139 (22.2)	38 (24.1)	78 (25.0)	23 (14.6)
Diabetes	33 (5.3)	21 (13.3)	12 (3.8)	0 (0)
Glomerulonephritis	136 (21.7)	34 (21.5)	75 (24.0)	27 (17.2)
Interstitial nephritis/pyelonephritis	51 (8.1)	11 (7.0)	21 (6.7)	19 (12.1)
Renal vascular disease	61 (9.7)	18 (11.4)	31 (9.9)	12 (7.6)
Other diseases	45 (7.2)	11 (7.0)	27 (8.7)	7 (4.5)
Unknown	102 (16.3)	24 (15.2)	51 (16.3)	27 (17.2)
Number of KTs, n(%)^a^				
1	540 (86.1)	133 (84.2)	263 (84.3)	144 (91.7)
>1	77 (12.3)	24 (15.2)	40 (12.8)	13 (8.3)
Donor type, n(%)^a^				
Living donor	376 (60.0)	102 (64.6)	212 (67.9)	62 (39.5)
Deceased donor	241 (38.4)	55 (34.8)	91 (29.2)	95 (60.5)
Mean (SD) BMI, kg/m^2^ ^a^	26.2 (4.6)	26.6 (4.5)	25.7 (4.3)	27.0 (5.4)
Comorbidities, n(%)^a^				
Diabetes Mellitus	97 (15.5)	31 (19.6)	47 (15.1)	19 (12.1)
Cardiovascular event	169 (27.0)	53 (33.5)	67 (21.5)	49 (31.2)
Cerebrovascular event	42 (6.7)	12 (7.6)	23 (7.4)	7 (4.5)
Immunosuppressants, n(%)^a^				
Prednisone	556 (88.7)	148 (93.7)	281 (90.1)	127 (80.9)
Tacrolimus	348 (55.5)	123 (77.8)	193 (61.9)	32 (20.4)
Mycophenolic acid	361 (57.6)	120 (75.9)	182 (58.3)	59 (37.6)

Data are presented as mean (SD) or median (IQR) for continuous variables and n (%) for categorical variables. Abbreviations: BMI, body mass index; IQR, interquartile range; KT, kidney transplantation; SES, socioeconomic status; SD, standard deviation.

a Variables with missing values: SES (0.8%), primary kidney disease (7.2%), number of KT (1.6%), donor type (1.6%), BMI (22.2%), diabetes (42.6%), cardiovascular event (39.1%), cerebrovascular event (47.8%), immunosuppressants (3.2%).

## Results

**FIGURE 1:**
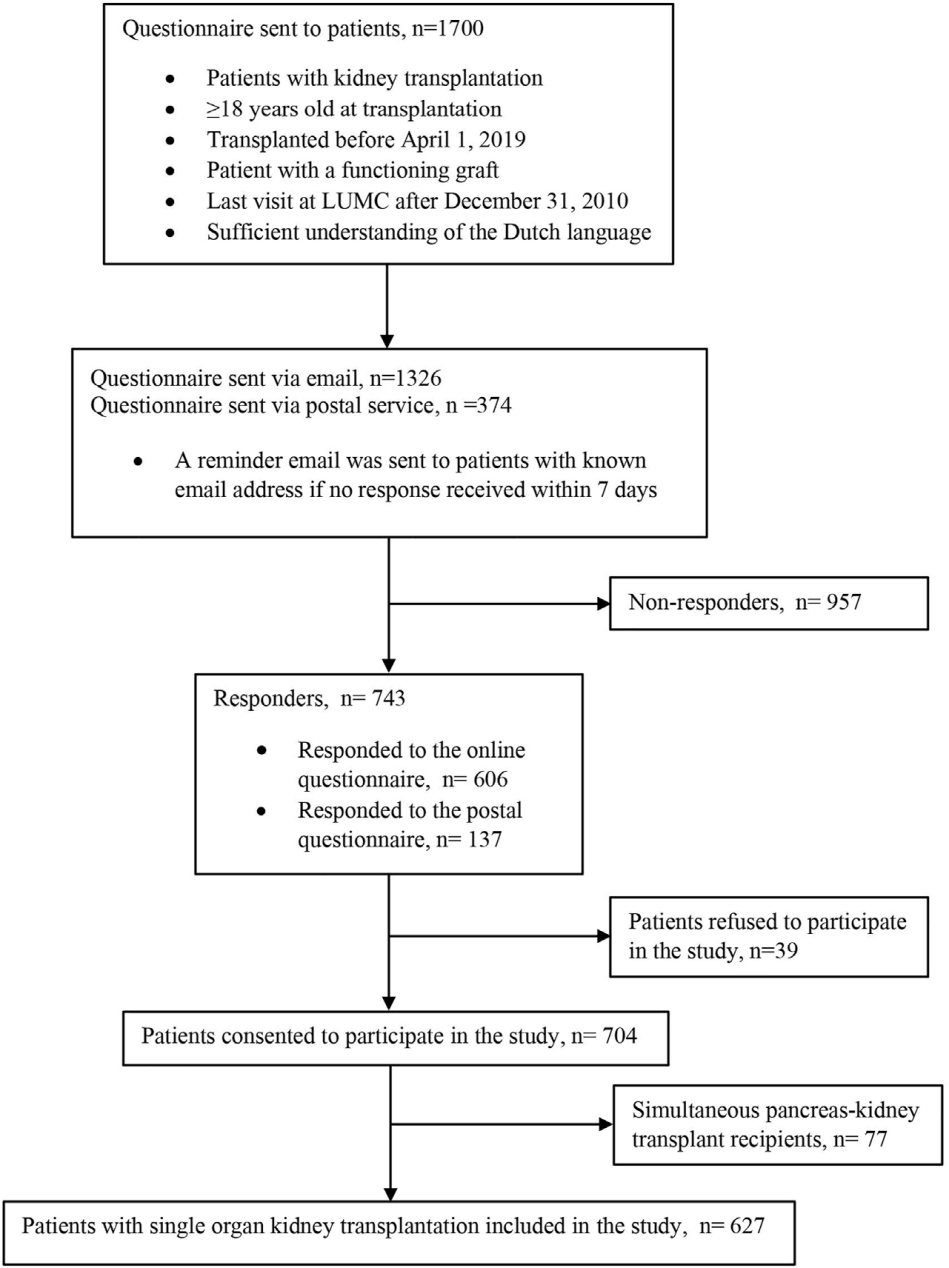
Flow chart of the study population.

Of the 1700 adult KTRs who were transplanted before 1 April 2019, at LUMC and met study inclusion criteria, 743 (43.7%) KTRs responded *via* email (*n* = 606) or *via* postal service (*n* = 137). 39 responders filled out the questionnaires but did not want to participate in this study. After excluding another 77 patients who received simultaneous pancreas-kidney transplantation, 627 KTRs were left to be included in the main analysis (Figure 1). Please see Supplementary Table S1 for the characteristics of the nonresponders.

### Patient Characteristics

Table 1 shows the sociodemographic and clinical characteristics of the responders in the total population and stratified by post-transplant time. The mean (SD) age of all included KTRs at study participation was 61.4 (11.3) years old; 93% of the KTRS were between 40 and 80 years old at the study. The median (IQR) post-transplant time was 9.0 (10.2) years, 74.5% of the KTRs had a partner, 89.8% had a medium or high SES, 57.4% received a high level of education, and 89.0% were employed, retired, or students. After stratification, KTRs with a post-transplant time of more than 15 years had the oldest age at study participation, the youngest age when receiving the transplantation, and the highest percentage of deceased donor kidney transplantation. KTRs with a post-transplant time of less than 5 years had the highest unemployment rate and the lowest percentage of living alone or being separated. Notably, the percentages of patients with diabetes as either PKD or comorbidity reduced as the post-transplant time increased. Difference in immunosuppressants was also observed in KTRs with different post-transplant time: patients with a post-transplant time of more than 15 years were less likely to receive prednisone, tacrolimus, and mycophenolic acid in comparison to the other two groups. Compared to the nonresponders, the study population had higher SES ranks and a lower percentage of diabetes as their PKD (Supplementary Table S1).

### Medication Nonadherence

Table 2 presents self-reported nonadherence to immunosuppressants in all study participants: 203 (32.4%) KTRs were identified as nonadherent based on the BAASIS-scoring algorithm. When focusing on the specific medication nonadherence domains, the results showed that nonadherence to *timing* (i.e., taking medication with more than 2 h difference from the prescribed time; 27.3%) was the most frequently reported nonadherent behaviour, followed by *issue with taking* (i.e., not take medication sporadically; 12.3%). Very few KTRs reported *drug holiday* (i.e., not take medication consecutively; 0.8%) or *dose reduction* (i.e., reduce the dosage of prescribed medication; 0.4%). Most nonadherent KTRs reported nonadherent behaviour once a month. After stratification by post-transplant time, the results showed that the proportion of nonadherent patients increased as the time after kidney transplantation increased overall and in the separate nonadherent behaviour domains.

**TABLE 2:** Medication nonadherence in the total study population and stratified by categories of post-transplant time.

Medication nonadherence, n (%)	Total (*n* = 627)	Post-transplant time			A “yes” to the question indicates
		<5 years (*n* = 158)	5–15 years (*n* = 312)	>15 years (*n* = 157)	
Medication nonadherence	203 (32.4)	43 (27.2)	105 (33.7)	55 (35.0)	Nonadherence to immunusuppressants in general^a^
Issues with taking	77 (12.3)	14 (8.8)	41 (13.1)	22 (14.0)	Not taken immunosuppressants some times in the past 4 weeks
Once a month	68 (10.8)	13 (8.2)	36 (11.5)	19 (12.1)	
More than once a month	9 (1.5)	1 (0.6)	5 (1.6)	3 (1.9)	
Drug holiday	5 (0.8)	1 (0.6)	2 (0.6)	2 (1.3)	Skipped several consecutive doses of immunosuppressants in the past 4 weeks
Once a month	3 (0.5)	1 (0.6)	2 (0.6)	0 (0)	
More than once a month	2 (0.3)	0 (0)	0 (0)	2 (1.3)	
Timing	171 (27.3)	35 (22.1)	88 (28.1)	48 (30.6)	Taken immunosuppressants with more than 2 h’ time difference from the prescribed dosing time in the past 4 weeks
Once a month	101 (16.1)	22 (13.9)	56 (17.9)	23 (14.6)	
More than once a month	70 (11.2)	13 (8.2)	32 (10.2)	25 (16.0)	
Dose reduction	2 (0.4)	0 (0)	1 (0.3)	1 (0.6)	Reduced the prescribed amount of immunosuppressants in the past 4 weeks
Once a month	1 (0.2)	0 (0)	1 (0.3)	0 (0)	
More than once a month	1 (0.2)	0 (0)	0 (0)	1 (0.6)	

a Any “yes” to the four questions of the four adherence-domains indicates medication nonadherence in general.

### Illness Perceptions

Mean (SD) scores of each illness perception are presented in Table 3. In general, the included KTRs believed to a relatively high extent that they understand their kidney disease (*illness coherence*) and that their kidney disease is a life-long chronic condition (*timeline*). They also had a strong belief that their treatment can control their disease (*treatment control*). The perceived *personal control* over their disease was lower than the perceived *treatment control* but could still be considered relatively high. The mean scores of the other illness perceptions laid around the midpoint of the scale (range: 3.8–5.0 on a 11-point scale ranging from 0 to 10), indicating that KTRs believed to a moderate extent that their kidney disease is a cause for concern (*concern*), has negative consequences upon their lives (*consequences*), and causes negative feelings (*emotional response*) and a high symptom burden (*illness identity*). After stratification, the results showed that KTRs with a longer post-transplant time believed to a lesser extent that their disease can be controlled by their treatment or by themselves (*treatment control* and *personal control*) and that they experienced a higher symptom burden due to kidney disease (*illness identity*).

**TABLE 3:** Illness perceptions of the total study population and stratified by categories of post-transplant time.

Illness perception, mean (SD)^a^	Total (*n* = 627)	Post-transplant time			A higher score indicates patients believe to a greater extent that…
		<5 years (*n* = 158)	5–15 years (*n* = 312)	>15 years (*n* = 157)	
Consequences	5.0 (2.9)	5.2 (2.9)	4.8 (2.9)	5.0 (3.1)	…their kidney disease has more negative consequences upon their life
Timeline	8.6 (2.7)	8.4 (2.9)	8.8 (2.6)	8.6 (2.7)	…their kidney disease lasts for a longer time
Personal control	3.8 (2.6)	3.4 (2.5)	3.8 (2.6)	4.3 (2.8)	…their kidney disease cannot be effectively controlled by themselves
Treatment control	2.2 (2.3)	1.7 (2.0)	2.2 (2.2)	2.7 (2.6)	…their kidney disease cannot be effectively controlled by their treatment
Illness identity	4.2 (2.9)	3.8 (2.8)	4.2 (2.9)	4.7 (2.9)	…their kidney disease causes more symptoms
Concern	4.7 (2.9)	4.7 (2.8)	4.7 (2.8)	4.9 (3.1)	…their kidney disease causes greater worries about their health
Illness coherence	1.6 (1.9)	1.7 (2.0)	1.3 (1.6)	1.9 (2.3)	…they do not understand their kidney disease
Emotional response	3.8 (2.9)	4.1 (3.1)	3.5 (2.9)	4.0 (2.9)	…their kidney disease causes more emotional distress

a Illness perceptions were measured on an 11-point scale ranging from 0 to 10, with higher scores reflecting stronger negative perceptions of their condition. *Personal control*, *treatment control* and *illness coherence* were recoded so that a higher score on these perceptions also indicate stronger negative illness perceptions.

### Illness Perceptions and Nonadherence to Immunosuppressants in KTRs

After adjusting for potential confounders, three illness perceptions (i.e., *illness identity*, *concern*, and *illness coherence*) were significantly associated with nonadherence to immunosuppressants in KTRs. More specifically, the results showed that with one increment in scores on the illness perceptions *illness identity*, *concern,* and *illness coherence*, the risk of nonadherence increased by 7%, 7%, and 11%, respectively (Table 4). For the other five domains (i.e., *consequences*, *timeline*, *personal control*, *treatment control,* and *emotional response*), the point estimates ranged from 1.02 to 1.05, indicating an association between less favourable illness perceptions of these illness perceptions and increased risk of medication nonadherence but with wider confidence intervals. Table 4 also shows the increased risk of medication nonadherence with every two increments in illness perception scores. None of the interactions between the separate illness perceptions and time after kidney transplantation were statistically significant (*p*-values ranged from 0.48 to 0.96).

**TABLE 4:** Associations between illness perceptions and medication nonadherence (*n* = 627).

Illness perception	Crude OR (95% CI)^b^	P-value	Adjusted OR (95% CI)^a^ ^,^ ^b^ per one increment in illness perception	Adjusted OR (95% CI)^a^ ^,^ ^c^ per two increments in illness perception	P-value	P-value for interaction^b^ (post-transplant time * illness perception)
Consequences	1.02 (0.97, 1.08)	0.44	1.02 (0.95, 1.08)	1.03 (0.91, 1.16)	0.64	0.48
Timeline	1.04 (0.98, 1.11)	0.21	1.02 (0.96, 1.10)	1.05 (0.91, 1.20)	0.51	0.96
Personal control	1.05 (0.99, 1.12)	0.10	1.05 (0.99, 1.13)	1.11 (0.97, 1.27)	0.12	0.52
Treatment control	1.05 (0.98, 1.23)	0.18	1.05 (0.97, 1.14)	1.11 (0.95, 1.29)	0.20	0.57
Illness identity	1.05 (0.99, 1.11)	0.14	1.07 (1.00, 1.14)	1.14 (1.00, 1.29)	0.05^d^	0.62
Concern	1.06 (1.00, 1.13)	0.04	1.07 (1.00. 1.14)	1.14 (1.00, 1.29)	0.05^d^	0.73
Illness coherence	1.08 (0.99, 1.17)	0.10	1.11 (1.01, 1.22)	1.23 (1.03, 1.48)	0.03	0.69
Emotional response	1.04 (0.98, 1.10)	0.22	1.03 (0.97, 1.10)	1.07 (0.94, 1.21)	0.32	0.64

Abbreviation: BMI, body mass index; CI, confidence interval; OR, odds ratio; SES, socioeconomic status.

a The adjusted variables included age at the study, sex, SES, rank, marital status, employment status, education level, primary kidney disease, comorbidities, BMI, donor type, time after kidney transplantation, the number of transplantations received, and immunosuppressants.

b OR, of one increment in illness perception scores on an 11-point scale.

c OR, of every two increments in illness perception scores on an 11-point scale.

d P-value < 0.05, namely: 0.045 for both illness perceptions “illness identity” and “concern”.

### Sensitivity Analyses

When repeating the logistic regression analysis without comorbidities and BMI (Supplementary Table S2), the results showed that, although the association between *illness identity* and *concern* and medication nonadherence became statistically insignificant, the ORs (95%CI) supported the results from the main analysis (i.e., *illness identity*: 1.06, 95%CI, 1.00 to 1.13, *p* = 0.06; *concern*: 1.06, 95%CI, 1.00 to 1.13, *p* = 0.06; *illness coherence*: 1.11, 95%CI, 1.02 to 1.22, *p* = 0.02).

## Discussion

Despite the improvements in nephrology care, adherence to immunosuppressants remains a challenge in KTRs. Our study detected nonadherence to immunosuppressants in a considerable proportion of prevalent Dutch KTRs and associations between negative illness perceptions and medication nonadherence to immunosuppressants.

The proportion of nonadherent KTRs in our study (32.4%) is similar to the results of a previous literature review, which also reported a high weighted mean prevalence (28%) of medication nonadherence to immunosuppressants in KTRs (5). However, the prevalence of medication nonadherence reported by different studies may not be directly comparable as their definition for medication nonadherence may differ. Regarding the nonadherence behavioural pattern, taking medication 2 h beyond the recommended dosing time was the most prevalent nonadherent behaviour in our study population (27.3%), followed by not taking their medication sporadically (12.3%). These findings are in line with other studies that also reported nonadherence behavioural patterns in KTRs (24, 25).

Furthermore, our results showed that stronger negative illness perceptions are associated with medication nonadherence to immunosuppressants in KTRs. More specifically, less understanding of kidney disease (*illness coherence*), greater worries about the kidney disease (*concern*), and experiencing more symptoms due to the kidney disease (*illness identity*) significantly increased the risk of medication nonadherence by 7%, 7%, and 11% with one unit increment on a 0-to-10 scale in our Dutch KTRs population. Our findings are in line with the results described by Cossart et al. that nonadherent KTRs believed to a lesser extent that they understand their kidney disease (*illness coherence*) (12). Additionally, our results indicated that the more worried patients were about their kidney disease (*concerns*), the more likely it was that they were nonadherent—an association that has also been reported in patients after myocardial infarction (26). A possible explanation for this finding is that highly concerned patients may have a more fatalistic attitude towards their disease (e.g., progression of their disease is inevitable) and are, therefore, less strict with their medication taking. Finally, our results showed that patients who attributed a greater symptom burden to their kidney disease were less adherent. This result is supported by findings reported by Rosenberger et al. (27) suggesting that KTRs with more adverse effect due to their chronic immunosuppressive treatment (e.g., tremor, diarrhoea, and fatigue) were more likely to be nonadherent. Of note, the results also suggested an association between less favourable illness perceptions of the other five domains (i.e., *consequences*, *timeline*, *personal control*, *treatment control*, and *emotional response*) and increased risk of medication nonadherence despite statistical insignificance.

In general, the association between illness perceptions and medication nonadherence is consistent with Leventhals’ CSM (6) and the results reported by others in patients with chronic conditions, such as hypertension and diabetes (28, 29). However, we did not observe the discrepancy found in the study conducted by Massey et al. (13), namely that some illness perceptions (*consequence* and *emotional response*) became more favourable over time while medication nonadherence still increased. The different study populations and study design may explain such differences in findings: Massey et al.’ population consisted of newly transplanted patients in a longitudinal study, while our study population was prevalent patients in a cross-sectional study. Notably, we did not detect a difference in the relationships between illness perceptions and medication nonadherence in patients with different time after kidney transplantation; however, we cannot rule out the possibility that these insignificant results are due to the participation of healthier KTRs regardless of their post-transplant time. Future studies with a longitudinal design and sufficient length of follow-up are needed to test the association between illness perceptions and medication nonadherence over time.

Our study suggests a need to improve medication adherence to immunosuppressants in KTRs along with previous research (5), and also suggests that negative illness perceptions could be a potential interventional target to achieve this. In our analyses, a perceived lack of understanding of kidney disease (*illness coherence*) was most strongly associated with medication nonadherence among other illness perceptions. However, a lack of illness understanding among patients is not uncommon in clinical practice: two previous studies in a clinical setting found that only 42% and 77% of the patients were able to list their diagnosis and that 14% and 17% of the patients were able to state the common side effects of their medication (30, 31). Such findings have shown adequate room to modify negative illness perceptions, which are indeed modifiable according to existing evidence in other patient groups and the CSM (6, 32–35). Current interventions to improve illness perceptions are mainly derived from the CSM framework and usually involve behaviour change techniques to modify the psychosocial determinants of unwanted (e.g., nonadherent) behaviour, such as patient education, motivational interviewing, goal setting, identifying and solving problems, improving social support, and facilitating support seeking (33, 34). In recent years, attempts have also been made to introduce self-management support programmes into care for patients with chronic conditions on top of the conventional treatment by healthcare professionals (35). Future studies are needed to facilitate translation of such knowledge into practice by identifying the effects of different behaviour change techniques to modify unhelpful illness perceptions, the efficient approaches to deliver such interventions to the patients, and the optimal logistics to implement such interventions into clinical practice. In addition to cognitive behavioural interventions, our results also suggested that patients could benefit from active management of immunosuppressant-related side effects in KTRs. Future studies may also focus on identifying potential risk factors for unhelpful illness perception to tailor intervention (e.g., age, gender, or SES). Finally, efforts are warranted to understand the clinically relevant level of occurrence and frequency of self-reported nonadherent behaviours in terms of the therapeutic effect of prescribed immunosuppressants to facilitate a more clinically relevant understanding of our results.

The strengths of this study include that our study population consists of KTRs covering a broad time span after kidney transplantation and that we are one of the first studies to examine the associations between illness perceptions and medication nonadherence in this specific population. Additionally, our analyses included a relatively large sample size, especially compared to the previous studies investigating similar topics (12, 13). Our study also has several limitations that should be taken into account. First, medication nonadherence was measured using self-report, which is prone to underestimate medication nonadherence (36). This could have potentially introduced outcome misclassification bias, leading to underestimating the association between illness perceptions and medication nonadherence. Second, the responders may not be representative of the general Dutch KTRs; compared to the nonresponders (Supplementary Table S1), responders were more likely to be in a better SES, receive living donor kidney transplantation, and were less likely to have diabetes as PKD. A previous survey study also suggests that responders better adhere to their medication regime than nonresponders (37). Such differences between responders and nonresponders could influence the generalizability of our results. Moreover, the majority of our study population was between 40 and 80 years old, which also limits the generalizability of our results. Third, our study was conducted in prevalent Dutch KTRs, and thus, future studies are needed to investigate whether our results can be generalized to different populations. Finally, due to our observational cross-sectional design, residual confounding as a result of unmeasured confounders (e.g., pill burden) exists and causal interpretation is limited, although the theoretical fundaments of CSM are considered quite robust (6, 38).

In conclusion, this study suggests that stronger negative illness perceptions are associated with medication nonadherence to immunosuppressants in KTRs. The high prevalence of medication nonadherence in our study indicates room for improvement and that KTRs need additional support to adhere to this strict medication regime. Targeting negative illness perceptions utilizing psychoeducational interventions could possibly optimize medication adherence and consequently improve health outcomes in KTRs. Future studies are needed to explore such interventions’ effects and identify facilitators and barriers for implementing such support strategies to help its uptake in clinical practice.

## Data Availability

The datasets presented in this article are not readily available because the data was collected for specific research purposes. Requests to access the datasets should be directed to AdV, A.P.J.de_Vries@lumc.nl.

## Abbreviation

BAASIS, the basel assessment of adherence to immunosuppressive medication scale; BMI, body mass index; CI, confidence interval; CSM, common sense model of self-regulation; EMERGE, the ESPACOMP medication adherence reporting guidelines checklist; IPQ, illness perception questionnaire; IQR, interquatile range; KT, kidney transplantation; KTRs, kidney transplant recipients; LUMC, Leiden university medical centre; OR, odds ratio; ORadj, adjusted odds ratio; PKD, primary kidney disease; SD, standard deviation; SES, socioeconomic status; STROBE, the strengthening the reporting of observational studies in epidemiology guideline; WMO, the medical research involving human subjects act.
