# Supplementary material for: Illness Perceptions and Medication Nonadherence to Immunosuppressants After Successful Kidney Transplantation: A Cross-Sectional Study
Source: Transpl Int. 2022 Feb 7;35:10073. doi: 10.3389/ti.2022.10073 (PMC8842226; doi:10.3389/ti.2022.10073)
Supplement: Supplementary file 1 [file DataSheet1.docx]

**Table S1**. Patient characteristics of responders and nonresponders (n=1449).

| **Characteristic** | **Responders**  (n=627) | **Non-responders**  (n=822) |
| --- | --- | --- |
| **Mean age at study (SD), yr** | 61.4 (11.3) | 58.3 (13.7) |
| **Mean age at KT (SD), yr** | 50.0 (13.1) | 48.0 (14.1) |
| **Median time after KT (IQR), yr** | 9.0 (10.2) | 7.9 (8.4) |
| **Female, n (%)** | 233 (37.2) | 316 (38.4) |
| **SES rank, n (%)^a^** |  |  |
| Low | 64 (10.2) | 188 (22.9) |
| Middle | 397 (63.3) | 481 (58.5) |
| High | 161 (25.7) | 138 (17.1) |
| **Primary kidney disease, n(%)^a^** |  |  |
| Congenital/hereditary kidney disease | 15 (2.4) | 43 (5.2) |
| Cystic kidney disease | 139 (22.2) | 106 (12.9) |
| Diabetes Mellitus | 33 (5.3) | 99 (12.0) |
| Glomerulonephritis | 136 (21.7) | 189 (23.0) |
| Interstitial nephritis/pyelonephritis | 51 (8.1) | 72 (8.8) |
| Renal vascular disease | 61 (9.7) | 72 (8.8) |
| Other diseases | 45 (7.2) | 58 (7.1) |
| Unknown | 102 (16.3) | 145 (17.6) |
| **Number of Tx, n(%)^a^** |  |  |
| 1 | 540 (86.1) | 717 (87.2) |
| >1 | 77 (12.3) | 102 (12.4) |
| **Donor type, n(%)^a^** |  |  |
| Living donor | 376 (60.0) | 433 (52.7) |
| Deceased donor | 241 (38.4) | 386 (47.0) |
| **Mean BMI (SD), kg/m^2 a^** | 26.2 (4.6) | 26.5 (4.7) |
| **Comorbidities, n(%)^a^** |  |  |
| Diabetes Mellitus | 97 (15.5) | 114 (13.9) |
| Cardiovascular event | 169 (27.0) | 182 (22.1) |
| Cerebrovascular event | 42 (6.7) | 51 (6.2) |
| **Immunosuppressants, n(%)^a^** |  |  |
| Prednisone | 556 (88.7) | 740 (90.0) |
| Tacrolimus | 348 (55.5) | 447 (54.4) |
| Mycophenolic acid | 361 (57.6) | 494 (60.1) |

Data are presented as mean (SD) or median (IQR) for continuous variables and n (%) for categorical variables. Abbreviation: BMI, body mass index; IQR, interquartile range; KT, kidney transplantation; SES, socioeconomic status; SD, standard deviation.

^a^ Variables with missing values: SES rank (0.8% and 1.8%), primary kidney disease (7.2% and 4.6%), number of transplantation (1.6% and 0.4%), donor type (1.6% and 0.4%), BMI (22.2% and 20.1%), diabetes (42.6% and 37.5%), cardiovascular event (39.1% and 37.3%), cerebrovascular event (47.8% and 42.6%) and immunosuppressants (3.2% and 3.4%) for responders and non-responders, respectively.

| **Illness perception** | **Crude OR (95% CI)^b^** | **P-value** | **Adjusted OR (95% CI)^a,b^** | **P-value** | **P-value for interaction^b^**  (post-transplant time * illness perception) |
| --- | --- | --- | --- | --- | --- |
| Consequences | 1.02 (0.97, 1.08) | 0.44 | 1.01 (0.95, 1.08) | 0.69 | 0.49 |
| Timeline | 1.04 (0.98, 1.11) | 0.21 | 1.02 (0.95, 1.09) | 0.59 | 0.94 |
| Personal control | 1.05 (0.99, 1.12) | 0.10 | 1.06 (0.99, 1.13) | 0.10 | 0.59 |
| Treatment control | 1.05 (0.98, 1.23) | 0.18 | 1.06 (0.98, 1.14) | 0.15 | 0.59 |
| Illness identity | 1.05 (0.99, 1.11) | 0.14 | 1.06 (1.00, 1.13) | 0.06 | 0.59 |
| Concern | 1.06 (1.00, 1.13) | 0.04 | 1.06 (1.00. 1.13) | 0.06 | 0.76 |
| Illness coherence | 1.08 (0.99, 1.17) | 0.10 | 1.11 (1.02, 1.22) | 0.02 | 0.62 |
| Emotional response | 1.04 (0.98, 1.10) | 0.22 | 1.03 (0.97, 1.09) | 0.35 | 0.66 |

**Table S2**. Associations between illness perceptions and medication nonadherence without adjusting for comorbidity and BMI (n=627).

Abbreviation: BMI, body mass index; CI, confidence interval; OR, odds ratio; SES, socioeconomic status.

^a^ The adjusted variables included age at study participation, sex, SES rank, marital status, employment status, education level, primary kidney disease, donor type, time after kidney transplantation, the number of transplantations received, and immunosuppressants.

^b^ OR of one increment in illness perception scores on an 11-point scale.
